# Using the intervention mapping protocol to develop a maintenance programme for the SLIMMER diabetes prevention intervention

**DOI:** 10.1186/1471-2458-14-1108

**Published:** 2014-10-27

**Authors:** Ellen BM Elsman, Joanne N Leerlooijer, Josien ter Beek, Geerke Duijzer, Sophia C Jansen, Gerrit J Hiddink, Edith JM Feskens, Annemien Haveman-Nies

**Affiliations:** Division of Human Nutrition; Academic Collaborative Centre AGORA, Wageningen University, P.O. Box 8129, 6700, VE Wageningen, The Netherlands; GGD Noord- en Oost-Gelderland (Community Health Service), P.O. Box 51, 7311, AB Apeldoorn, The Netherlands; Strategic Communication, Sub-department Communication, Philosophy and Technology: Centre for Integrative Development, Social Sciences, Wageningen University, P.O. Box 8130, 6700, EW Wageningen, The Netherlands

**Keywords:** Type 2 diabetes mellitus, Prevention, Behaviour maintenance, Physical activity, Dietary behaviour, Intervention mapping

## Abstract

**Background:**

Although lifestyle interventions have shown to be effective in reducing the risk for type 2 diabetes mellitus, maintenance of achieved results is difficult, as participants often experience relapse after the intervention has ended. This paper describes the systematic development of a maintenance programme for the extensive SLIMMER intervention, an existing diabetes prevention intervention for high-risk individuals, implemented in a real-life setting in the Netherlands.

**Methods:**

The maintenance programme was developed using the Intervention Mapping protocol. Programme development was informed by a literature study supplemented by various focus group discussions and feedback from implementers of the extensive SLIMMER intervention.

**Results:**

The maintenance programme was designed to sustain a healthy diet and physical activity pattern by targeting knowledge, attitudes, subjective norms and perceived behavioural control of the SLIMMER participants. Practical applications were clustered into nine programme components, including sports clinics at local sports clubs, a concluding meeting with the physiotherapist and dietician, and a return session with the physiotherapist, dietician and physical activity group. Manuals were developed for the implementers and included a detailed time table and step-by-step instructions on how to implement the maintenance programme.

**Conclusions:**

The Intervention Mapping protocol provided a useful framework to systematically plan a maintenance programme for the extensive SLIMMER intervention. The study showed that planning a maintenance programme can build on existing implementation structures of the extensive programme. Future research is needed to determine to what extent the maintenance programme contributes to sustained effects in participants of lifestyle interventions.

**Electronic supplementary material:**

The online version of this article (doi:10.1186/1471-2458-14-1108) contains supplementary material, which is available to authorized users.

## Background

Studies have shown that lifestyle interventions show promising results in reducing the risk of type 2 diabetes mellitus (T2DM) in high-risk individuals. A meta-analysis on the effects of lifestyle interventions on T2DM incidence showed that risk reductions ranged from 30% to 67% in the lifestyle education intervention group compared with the control group [1]. Intervention trials, such as the Finnish Diabetes Prevention Study (DPS), the American Diabetes Prevention Program (DPP), and the Dutch Study on Lifestyle Intervention and Impaired glucose tolerance Maastricht (SLIM), have shown that the progress to T2DM can be prevented or postponed with risk reductions of 47-58% [2–5]. Although lifestyle interventions have shown to be effective in reducing the risk for T2DM, maintenance of achieved results is difficult, as participants often experience relapse after the intervention has ended [6–14]. The trans theoretical model (TTM) and stages of change [15] state that individuals can relapse at each stage of behaviour change, including the maintenance stage. Research suggests that maintenance of behaviour change in lifestyle interventions requires more attention [7]. Lifestyle interventions, therefore, can be complemented with a maintenance programme, whereby a less intensive form of professional support is provided to the participants [16]. Maintenance programmes of the DPS and the DPP showed that risk reductions achieved during the extensive intervention could be sustained in the long-term, with diabetes incidence reductions in the lifestyle group of 38% and 34% respectively, compared with the control group [17, 18]. These studies were performed in an experimental setting in which the conditions are strictly controlled to secure high internal validity. However, limited research is available regarding effects of maintenance programmes implemented in a real-life setting, in which conditions are less strictly controlled and resemble everyday real-life. Moreover, the experimental studies on maintenance programmes have focussed on reporting outcomes rather than describing programme development and implementation [19–21]. Consequently, effective working mechanisms of maintenance programmes are difficult to identify [22]. Information on these mechanisms would facilitate the planning and implementation of maintenance programmes elsewhere. Therefore, this paper describes the systematic development of a maintenance programme for the SLIM iMplementation Experience Region Noord- en Oost-Gelderland (SLIMMER), implemented in Dutch primary health care, using the Intervention Mapping (IM) protocol [23].

### The SLIMMER intervention

To investigate whether diabetes prevention interventions implemented in real-life settings could achieve similar effects as interventions in experimental settings, the Dutch proven effective experimental SLIM study was translated into the SLIMMER intervention, which was implemented in a Dutch real-life setting [24, 25]. SLIMMER is a diabetes prevention intervention for 40–70 years old individuals at high risk for developing T2DM who were living in Apeldoorn and Doetinchem, two medium-sized cities in the Netherlands. Individuals with fasting plasma glucose between 6.1-6.9 mmol/L or an increased risk of diabetes were referred to SLIMMER by their general practitioners. Participants of the SLIMMER intervention received a combined physical activity and nutritional intervention for ten months, delivered by primary health care professionals. The physical activity intervention consisted of weekly group-based exercise sessions guided by a physiotherapist. For the nutritional intervention, participants participated in 5–8 individual sessions and one group session with a dietician [26]. Details of the intervention programme are described elsewhere [25–27]. A pilot study of the SLIMMER intervention was conducted initially to confirm feasibility and likelihood of desired impact [27]. Subsequently, the SLIMMER intervention was implemented on a larger scale in a randomized controlled trial (RCT) which included 316 participants (155 in intervention group and 161 in control group). For the remainder of this paper, we refer to the larger RCT of the SLIMMER intervention as the ‘extensive programme’.

## Methods

### Intervention mapping

IM describes the process from problem identification to problem solving through planning theory- and evidence-based health promotion interventions [23]. The IM protocol consists of six steps: 1) conduct a needs assessment, 2) formulate change objectives, 3) select theoretical methods and practical applications, 4) produce programme components and materials, 5) design an implementation plan, and 6) design an evaluation plan [23]. IM is characterised by three perspectives that are applied in each step: participation of all relevant stakeholders in intervention planning [28], using theory and evidence, and using an ecological approach, meaning that all relevant individuals, groups and organisations that are related to the health problem are considered as target groups or implementers of the intervention [29]. In this paper we describe how IM steps 1–5 were applied in the planning of the SLIMMER maintenance programme. Step 6, designing an evaluation plan has been described by Duijzer et al. [26]. The maintenance programme will be evaluated together with the extensive intervention. The current paper adheres to RATS standards of reporting of qualitative data. The WU Medical Ethics Committee approved the study protocol and all subjects gave their written informed consent before the start of the study.

#### Step 1: Needs assessment

In the first step of the IM protocol (needs assessment), the health problem is analysed, followed by an exploration of related behaviours, environmental factors, and behavioural determinants [23]. In our study, the needs assessment included a literature study exploring theories and determinants of maintained behaviour. Results of this literature study informed the design of focus group guides, used in the focus group discussions (FGDs). The aim of the FGDs was to supplement results of the literature study, exploring the determinants of maintained behaviour (healthy diet and physical activity pattern) and the needs of participants in lifestyle maintenance interventions. In addition, suggested components, opportunities, barriers and methods for adoption and implementation of the SLIMMER maintenance programme were explored. The method of FGDs was selected because of the expected added value of group interaction and generation of ideas. The needs assessment informed the design of the SLIMMER maintenance programme and the adoption and implementation plan.

### Literature study

A literature study was conducted to explore important determinants of maintained behaviour and habitual behaviour using the databases ‘PubMed’ , ‘Scopus’ and ‘Web of Science’. Search terms included ‘behaviour’ , ‘maintenance’ , ‘habits’ , ‘relapse prevention’ , ‘weight loss’ , ‘diabetes prevention’ , ‘nutrition’ , ‘physical activity’ , and combinations of these terms. In addition, reference lists of articles were used to identify other relevant studies.

### Focus group discussions (FGDs)

Three FGDs were conducted to identify barriers and facilitators of maintaining a healthy lifestyle, potential components in the maintenance programme, and opportunities and barriers for adoption and implementation of these components. The FGDs were conducted with representatives of sports clubs in Apeldoorn (n = 9), participants of the SLIMMER intervention in Apeldoorn (n = 7) and physiotherapists and dieticians in Apeldoorn and Doetinchem (n = 6). Involving those stakeholders in programme development might potentially lead to more effective and innovative programmes with more sustainable effects [30, 31]. In addition, it was expected that their involvement in the FGDs would increase their motivation and sense of ownership, enhancing programme acceptability and effectiveness [32]. FGD participants were selected on a voluntary basis for feasibility reasons.

The first FGD was conducted with chairpersons and/or secretaries representing the seven local sports clubs involved in the maintenance programme. Chairpersons and/or secretaries were personally invited. When chairpersons were not able to attend (n = 2), secretaries were approached. Of three sports clubs, both the secretary and the chairperson attended. One sports club was not able to participate, so in total, four chairpersons (3 male, 1 female) and five secretaries (2 male, 3 female) of six local sports clubs participated in the FGD.

The second FGD was conducted with a selection of SLIMMER participants in Apeldoorn (n = 7). Participants in Apeldoorn (n = 131) were invited via the SLIMMER newsletter. Five participants responded to this invitation, of which three were able to attend. These persons were 3–5 months from completion of the extensive intervention. In addition, four participants of the SLIMMER pilot study with experience in maintaining a healthy lifestyle (18 months after phasing out of the SLIMMER pilot study) were personally invited. In total, seven (former) SLIMMER participants (3 male, 4 female) participated in the FGD.

The third FGD was conducted with physiotherapists (n = 16, clustered in 9 physiotherapist practices) and dieticians (n = 11, five of them were employed by a home care organisation, six were self-employed) of the SLIMMER intervention. All implementers of the SLIMMER intervention were personally invited to participate. Four physiotherapists (1 male, 3 female, all from different practices) and two dieticians (both female, one associated with a home care organisation, one self-employed) were willing to participate. Both physiotherapists and dieticians were organised in local professional networks, where they regularly discuss subjects that have been addressed during the FGD.

Focus group guides were developed to facilitate the FGDs, including questions on barriers and facilitators of maintaining a healthy lifestyle; expected needs of SLIMMER participants after the extensive intervention; suggested components for the maintenance programme of the SLIMMER intervention; and implementation methods, opportunities and barriers for these suggested components.

All FGDs lasted between 60 and 90 minutes (mean: 77 minutes). A trained moderator (JtB) guided the FGDs and a research assistant (EE) took notes. All FGDs were tape-recorded after obtaining informed consent from the participants and were subsequently transcribed. Data were analysed using a general inductive approach [33]: transcripts were read several times by the first author and coded into topics, until themes emerged. Overlap and redundancy among themes was reduced, leaving broader themes. To integrate results of FGDs and literature review, themes derived from the FGDs were linked to determinants of maintained behaviour found in literature.

#### Step 2: Formulating change objectives

Objectives for the maintenance programme were specified in the second step of the IM protocol [23]. Subsequently, performance objectives were identified for each behavioural outcome, describing the sub-behaviours that have to be accomplished to achieve behavioural outcomes.

Specific change objectives were formulated by linking performance objectives to determinants that were identified in the needs assessment. The change objectives describe what participants are expected to know, think or do as a result of participation in the maintenance programme, for example ‘Participants demonstrate that they can set realistic targets and comply with these targets’. The result of this process was a matrix of change objectives detailing what would be addressed in the maintenance programme.

#### Step 3: selecting theoretical methods and practical applications

In IM step 3, theoretical methods and practical applications were selected. A theoretical method is a general technique or process which is derived from theory and can be applied to influence behavioural determinants. A practical application is a specific application of a theoretical method, adjusted to the intervention setting, tailored to the target population, and applied considering parameters for effective use of methods [23]. For each behavioural determinant, appropriate theoretical methods were identified from literature [22]. These theoretical methods were translated into practical applications that were suitable for the maintenance programme, taking into account the needs of SLIMMER participants. To determine applicability in the programme, applications were discussed with the SLIMMER project group and the intended implementers of the maintenance programme.

#### Step 4: Producing programme components and materials

In the fourth step of IM, the maintenance programme was developed. Based on needs of participants, feasibility of implementation, and resource constraints, change objectives and applications were selected from a large list to be addressed in the programme. Applications were clustered to form programme components. A detailed manual describing the intervention components and programme materials was developed. Due to time limitations as a result of working in the real-life setting, programme activities and materials were not pre-tested.

#### Step 5: Designing an implementation plan

In step 5 programme adoption, implementation and sustainability were considered [23]. In this step, step 2 and 3 of the intervention mapping protocol were repeated, to identify the required behaviours of the implementers. Results of the FGDs (IM step 1) were used to develop an adoption and implementation plan. Key components of this step included production of implementation plans and a meeting with implementers to discuss adoption and implementation of the maintenance programme.

## Results

### Step 1: Needs assessment

#### Literature study

In the literature study determinants of maintained behaviour and habitual behaviour were explored. To ensure that the healthy behaviour becomes habitual, the behaviour needs to be repeated [34]. The TTM is a model that has often been used in behaviour change research and interventions [15], amongst others in the effective SLIM study [25, 35] on which SLIMMER is based. It was decided to use the TTM to tailor the maintenance programme to the stage of change participants are in, a purpose for which the model is frequently used [15, 36].

According to the TTM, people progress through a series of stages when they change their behaviour [15]. Participants who have participated in the extensive SLIMMER intervention are likely to be in the action phase. In order to progress from action to maintenance, participants particularly need to be supported with regard to attitude, subjective norms and perceived behavioural control. These determinants have been described by the Theory of Planned Behaviour (TPB) [37] and predict people´s behavioural intention [38] and maintained behaviour change [39, 40].

Regarding maintained exercise behaviour in older adults, multiple studies found that attitude, perceived behavioural control and subjective norm are important predictors [41, 42]. Subjective norm and perceived behavioural control seem to be important predictors with respect to nutrition behaviour [43]. However, the importance of determinants with respect to behaviour maintenance differs. Several studies found that perceived behavioural control is likely to be more important than attitude [38, 44], and reducing actual barriers might be an important strategy in behaviour maintenance. Furthermore, behaviour will be better maintained when participants’ experiences of autonomy, competence and relatedness are enhanced [45]. In addition to individual-level determinants, research suggest that family, work, study and neighbourhood environment may be important factors influencing behaviour maintenance [46], indicating that the social-cultural environment is more important than the physical environment. However, evidence on environmental determinants is limited because of lacking high-quality studies and study replications [47].

#### Focus group discussions

The FGDs with (former) SLIMMER participants, representatives of sports clubs, and representatives of physiotherapists and dieticians resulted in the identification of inhibiting and facilitating factors to maintain a physical activity pattern and a healthy diet for SLIMMER participants, as well as suggestions for the maintenance programme.

Regarding physical activity, many (former) SLIMMER participants mentioned lack of confidence to join a sports club or gym as an important barrier to continue physical activity, which was confirmed by representatives of sports clubs and literature [48]. Other important barriers for physical activity mentioned by all groups of respondents were physical complaints, lack of motivation and financial constraints. These barriers are also often reported in literature [48–51]. On the other hand, the social aspects of sporting together were mentioned in all FGDs as an important facilitating factor to continue physical activity. Other facilitating factors that were frequently mentioned were feeling healthier, being motivated to prevent T2DM, receiving social support from family and friends, and receiving guidance when exercising.

With regard to maintenance of a healthy diet in the FGDs, most of the (former) SLIMMER participants expected this would be easier than maintaining physical activity levels. This was confirmed by participants in the FDG with physiotherapists and dieticians and by other studies [52–55]. SLIMMER respondents thought they had sufficient knowledge and skills to maintain a healthy diet, although two respondents mentioned they lacked creativity to cook healthy and tasty dishes. Respondents mentioned they were able to handle tempting eating situations independently, as they had received sufficient advice to resist these situations.

Finally, the FGDs provided insight in respondents’ opinions regarding necessary components of the maintenance programme. SLIMMER respondents particularly preferred to receive support to maintain physical activity. In all FGDs, respondents mentioned that introducing SLIMMER participants to local sports clubs was regarded as a useful component of the maintenance programme, as this would help to reduce the threshold for participants. Furthermore, SLIMMER respondents expressed that they would like to receive information about cooking clubs, as this could support them to become more creative in composing healthy dishes. In addition, physiotherapists and dieticians mentioned that a concluding meeting with SLIMMER participants could help participants to focus on maintenance of a healthy lifestyle, as they noticed that a majority of participants did not prepare themselves for the period after the extensive intervention. In addition, SLIMMER respondents, physiotherapists and dieticians proposed to organise a return session a few months after the extensive intervention. In this session the maintenance of a healthy lifestyle could be discussed. SLIMMER respondents also mentioned they would like to receive a blood glucose test one year after the extensive intervention to determine whether their glucose levels have improved or stabilized.

Concluding, the literature study and FGDs showed that determinants of the TPB are important in behaviour maintenance, although perceived behavioural control and self-efficacy seem to be more important than attitude. Even though environmental determinants are also likely to influence behaviour maintenance, evidence is limited. Reducing actual barriers might be an important strategy to maintain the healthy behaviour in participants, as well as continuation of support. Furthermore, behaviour might be better maintained when participants’ experiences of autonomy, competence and relatedness are enhanced.

### Step 2: Formulating change objectives

The behavioural outcomes of the maintenance programme were: ‘Participants maintain the acquired healthy diet and physical activity pattern independently’. The performance objectives of the two behavioural outcomes were based on the needs assessment in IM step 1, Dutch dietary guidelines [56] and Dutch healthy physical activity norms [57]. The performance objectives are described in Table 1. The literature study and FGDs in IM step 1 resulted in the identification of determinants of maintained and habitual behaviour, used to formulate change objectives [15, 37, 58, 59]. Furthermore, knowledge was selected as a determinant, as it is a prerequisite for instigating behaviour change and other behavioural determinants including attitude, subjective norm and perceived behavioural control [23, 60–62]. Even though evidence suggests that attitude is of minor importance for behaviour maintenance, it was decided to include attitude as a determinant. Participants need a positive attitude towards the new behaviour to maintain their healthy lifestyle independently, and towards new activities offered to them. In addition, limited evidence suggests that environmental determinants are related to behaviour maintenance. However, there was a large variation among participants in the SLIMMER maintenance programme with regard to their social (work, study, neighbourhood, and family) environment. It was therefore practically not feasible to include determinants aimed at environmental change. Because the selected determinants are also important in other stages than maintenance [63], participants who have relapsed might benefit from the maintenance programme as well. The behavioural determinants were used in the matrices of change objectives in IM step 2. Intervention developers specified change objectives for each determinant, linking it to the performance objective. The change objectives were discussed with researchers until consensus was reached. Examples of change objectives are presented in Table 2. The complete matrices of change objectives are shown in Additional file 1.

**Table 1:** **Behavioural outcomes and performance objectives for participants of the SLIMMER maintenance programme**

Behavioural outcomes	Performance objectives
1. SLIMMER participants maintain the acquired healthy diet independently	1.1 Comply with Dutch dietary guidelines
	1.2 Create social support to maintain healthy diet
	1.3 Identify situations that could be tempting to relapse
	1.4 Compose action plans with realistic targets to maintain healthy diet
	1.5 Maintain monitoring of weight and diet
2. SLIMMER participants maintain the acquired healthy physical activity pattern independently	2.1 Comply with Dutch norm for healthy physical activity
	2.2 Create social support to maintain healthy physical activity pattern
	2.3 Identify situations that could be tempting to relapse
	2.4 Compose action plan with realistic targets to maintain healthy physical activity pattern
	2.5 Maintain monitoring of physical activity pattern

**Table 2:** **Examples of change objectives for the SLIMMER maintenance programme**

Behavioural outcome: SLIMMER participants maintain the acquired healthy diet independently				
Performance objective:	*Behavioural determinants*			
	Knowledge	Attitude	Subjective norm	Perceived behavioural control
Comply with the Dutch dietary guidelines	Describe Dutch guidelines for healthy diet;	Emphasize importance of a healthy diet	List other participants or persons from social environment who comply to guidelines healthy diet;	Express confidence in handling negative social and environmental stimuli and obstructive thoughts which complicate compliance to guidelines healthy diet
	Explain why complying to Dutch guidelines healthy diet is important		Mention the support they receive from their social environment when complying to guidelines healthy diet	
**Behavioural outcome: SLIMMER participants maintain the acquired healthy physical activity pattern independently**				
**Performance objective:**	***Behavioural determinants***			
	**Knowledge**	**Attitude**	**Subjective norm**	**Perceived behavioural control**
Compose action plan with realistic targets to maintain healthy physical activity pattern	Explain importance of setting targets	Convince others that setting targets is important	List other participants or persons from social environment who have an action plan to be physically active;	Demonstrate that they can set realistic targets and comply to these targets
			Mention the support they receive from their social environment when composing an action plan	

### Step 3: Selecting theoretical methods and practical applications

After careful consideration of parameters for use, theoretical methods and practical applications addressing the determinants were selected to address the change objectives (IM step 2). Researchers composed a preliminary list of possible theoretical methods, which were discussed with intervention developers. Researchers and intervention developers translated the selected theoretical methods into practical strategies in a joint meeting. Results from the FGDs informed the translation of theoretical methods into practical applications, ensuring that the needs of the SLIMMER participants were taken into account. Although the maintenance programme focuses on the transition of participants from action to maintenance, theoretical methods and practical applications were also selected for participants who have relapsed. Furthermore, theoretical methods and practical applications were selected to maximise participants’ experienced autonomy, competence and relatedness, to offer continuation of support and to reduce barriers to maintain the behaviour. The selected practical applications were shortly discussed with the implementers of the maintenance programme, to assess applicability and feasibility. When necessary, small changes were made, resulting in applications which are easier to implement. In Table 3 examples of methods and applications are described. One of the selected methods is ‘goal setting’, which relates to the performance objective ‘Compose an action plan with realistic targets to maintain a healthy physical activity pattern’. In the practical application of this method, participants set targets and make an action plan during the concluding meeting whereby participants have to be committed to the goal and that the goal is challenging, but achievable (parameters for use) [23]. The determinants, their linked theoretical methods and practical strategies used in the maintenance programme are shown in Additional file 2.

**Table 3:** **Examples of theoretical methods and practical applications for behavioural determinants**

Behavioural Determinant	Theoretical method	Definition [23]	Parameters for use	Practical application
Knowledge	Advance organizers	Presenting an overview of material that enables the learner to activate relevant schemas so that new material can be associated	Schematic representations of content or guides to what is to be learned	Providing an online overview of activities of local facilitators of physical activity and healthy nutrition on SLIMMER website
Attitude	Elaboration	Stimulating the learner to add meaning to the information that is processed	Individuals with high motivation and cognitive ability	During the return session, participants discuss how they feel about their behaviour change
Perceived behavioural control	Self-monitoring of behaviour	Prompting the person to keep a record of specified behaviours	The monitoring must be of the specific behaviour; the data must be interpreted and used; the reward must be reinforcing to the individual	During concluding meeting, the importance of monitoring is explained and methods to monitor behaviour are provided. The importance of self-monitoring can be highlighted again during return session
Perceived behavioural control	Goal setting	Prompting planning what the person will do, including a definition of goal-directed behaviours that result in target behaviour	Commitment to goal; goals that are challenging but achievable within the individual’s skill level	During concluding meeting, participants set targets and make an action plan, which is added to the personal file of the participant
Habits	Implementation intentions	Prompting making if-then plans that link situational cues with responses that are effective in attaining goals or outcomes	Existing positive intention	Participants receive an action plan in which they formulate specific goals and ways to achieve them. Feedback is given during the return session

### Step 4: Producing programme components and materials

Practical applications were clustered in three main programme components and six minor components. The main components are: conduct sports clinics at local sports clubs, perform a concluding meeting with physiotherapist and dietician and visit a return session with physiotherapist, dietician and physical activity group. The six minor components are: refer to SLIMMER and behaviour maintenance by the general practitioner, offer weighing and measuring at the general practitioner’s practice, receive SLIMMER newsletter from project group, visit SLIMMER website for tips, advices and sports clubs, offer contacting the project group through SLIMMER email and phone. The limited financial resources determined the selection of programme components of the maintenance programme. Even though participants suggested to include a blood glucose test after one year, this could not be included due to financial limitations. The content of the nine components was based on methods and applications identified in IM step 3.

The first main programme component consists of sports clinics organised by local sports clubs. Participants are invited to participate twice in three sports clinics of their preference to reduce barriers to join a sports club. The sports clinics aim to let participants experience that sporting is fun and that they are able to perform the particular sport, thereby increasing their sense of autonomy and competence. In addition, their feeling of relatedness will be enhanced because of guidance from competent trainers and their physiotherapists. The sports clinics take place during the last two months of the extensive SLIMMER intervention. The second main component is a concluding meeting with a physiotherapist and a dietician to get prepared for the period after SLIMMER. Participants set realistic targets, are advised how they can monitor their behaviour with the SLIMMER action plan and a food diary, and are informed what to do in case of a relapse. The concluding meeting takes place during the last two weeks of the extensive SLIMMER intervention. The third main component is a return session with the physiotherapist, dietician and physical activity group which is organised three months after the extensive SLIMMER intervention ended. During the return session, participants reflect on the SLIMMER intervention, the period after the extensive intervention, and behaviour maintenance. Participants are advised on how to continue a healthy diet and physical activity pattern and are weighed and measured. When participants are relapsed, they are motivated to take up their healthy lifestyle again, and are offered tips and tools to do so.

The six minor components of the maintenance programme are less intensive and based on existing applications of the extensive SLIMMER intervention. These components are offered individual to SLIMMER participants by the general practitioner or SLIMMER project group, who were involved in the design of the extensive intervention and maintenance programme. The minor components are planned to be systematically delivered at the end of the extensive SLIMMER intervention, up to one year after the extensive intervention has ended.

### Step 5: Designing an implementation plan

The aim of step 5 was to anticipate from the start on programme adoption, implementation, and sustainability. The intended implementers of the maintenance programme were physiotherapists, dieticians and general practitioners who were involved in the implementation of the extensive SLIMMER intervention. New implementers were trainers of local sports clubs. The behavioural outcomes for physiotherapists, dieticians and general practitioners included: 1) to provide support to SLIMMER participants to maintain their healthy diet and physical activity pattern, and 2) to encourage SLIMMER participants to take initiative and responsibility for their own health. The behavioural outcome for local sports clubs was to support SLIMMER participants in maintaining their healthy physical activity pattern. All implementers receive training and instructions to implement the maintenance programme.

Step 5 resulted in the development of a schedule for conducting sports clinics and manuals for physiotherapists, dieticians and general practitioners. Manuals include the intervention materials as well as step-by-step instructions on how to implement components of the maintenance programme, and a detailed time table. The manuals are introduced to the implementers in a 30-minute instruction meeting. Trainers of sports clubs are individually instructed by the SLIMMER project group. Overall, support throughout the implementation of the maintenance programme is offered by the SLIMMER project group through email and telephone.

## Discussion

This paper describes the development of a maintenance programme for the SLIMMER diabetes prevention intervention, which was guided by IM [15]. The aim of the maintenance programme is to support a maintained healthy diet and physical activity pattern of SLIMMER participants, acquired during the extensive SLIMMER intervention. This support is provided by offering sports clinics, a concluding meeting, and a return session to the participants. In addition, participants receive reminders and are offered various opportunities to contact professionals when needed.

The TTM was used to tailor the maintenance programme to the stage of change of participants. The assumption of the maintenance programme is that participants are likely to be in the action phase, as they have been enrolled in the extensive intervention for at least eight months. However, this was not measured before their enrolment in the maintenance programme. One of the criticisms of the TTM is that participants are often misclassified in stage-based interventions [64, 65]. To address the risk of misclassification of participants, the programme also addresses determinants that are relevant for participants who have relapsed to a previous stage (e.g. knowledge and attitude) [63]. Furthermore, evidence is in favour regarding the effectiveness of stage-based interventions over non-stage-based interventions, although the effects on long-term behaviour change requires further research [66].

The needs assessment contributed to the programme’s relevance by integrating the needs and suggestions of participants for the maintenance programme [67]. Because the literature study was not performed systematically due to limited time, it cannot be ensured all relevant literature is taken into account. However, FGDs were used to supplement the information from literature, thereby increasing the validity of the needs assessment [68]. The maintenance programme does not involve and intervene in the social environment of the participants. This may limit the programme’s effectiveness, since the importance of the direct social environment in maintenance of health behaviour was noted in the FGD with SLIMMER participants and literature [47, 69, 70]. Another limitation was the low response of SLIMMER participants to participate in the FGDs. This may have resulted in overlooking specific needs and wishes of non-responders, which could be different from the needs and wishes of the FGD participants, who may have higher motivation levels. With respect to the FGD with physiotherapists and dieticians, it was believed that most opinions were taken into account, because of their organisation in professional local networks. However, with one FGD per group of stakeholders, data saturation cannot be ensured. Due to time limitations, it was not possible to organise additional FGDs.

IM was found to be useful in ensuring that all important objectives were addressed in the maintenance programme, and by selecting theoretical methods and practical applications that would contribute to these objectives.

To our knowledge, this is the first paper that describes the systematic development of a lifestyle maintenance programme in a real-life setting. The maintenance programmes of the DPP and DPS had a longer duration than the SLIMMER maintenance programme. The DPP had yearly and six-monthly outcome assessment examinations for approximately 7 years [18], whereas the DPS had yearly, and after five years biyearly, outcome assessment examinations for a median time of 7 years [17]. The maintenance programme of the DPP was more intensive than the SLIMMER maintenance programme as well, offering a 16-session DPP-like lifestyle programme, followed by three-monthly lifestyle sessions and two group classes comprising four sessions every year [18]. However, the maintenance programme of the DPS is not as intensive as the SLIMMER maintenance programme, since no intervention was delivered during the 7-year follow-up period [17].

Maintenance programme planning is different from intervention planning for two reasons. First, maintenance programmes build upon extensive interventions, which usually already have a defined target population, programme implementers, and project structures such as a project group. Other studies reported that systematic planning of interventions had been rather time-consuming [19, 71]. In the planning of the maintenance programme this was experienced to a lesser extent because the majority of implementers were already involved, and part of the materials, methods and applications had been developed for the extensive intervention. The short timespan experienced in the real-life setting also introduced new challenges in adhering to the IM protocol. For example, programme materials were not pretested with implementers and participants.

A second difference between regular and maintenance programme planning is that maintenance programmes aim to maintain the acquired behaviour and are therefore likely to focus more on skills and reducing the barriers to maintain behaviour. In this research, offering tools and guidance to maintain the healthy lifestyle and reducing barriers to maintain physical activity levels was particularly important.

Participants of lifestyle interventions often experience relapse after the intervention has ended [6–14]. Therefore, it is suggested that lifestyle interventions should focus more on behaviour maintenance once healthy behaviour is initiated [72]. The present study described the systematic development of a maintenance programme for the SLIMMER intervention. This maintenance programme was not fully integrated with the extensive intervention, as it was developed separately. In future, programme planners should incorporate a maintenance component when developing lifestyle interventions [52, 73]. This might lead to more effective programmes of which the content of the extensive programme and maintenance programme are fully connected.

Compared to behaviour change, less is known about behaviour maintenance, making it difficult to identify effective working mechanisms from literature. The maintenance programmes of the DPS and DPP have indicated that effects of diabetes prevention interventions can be maintained [18, 74], but the development of these programmes is not fully described in literature [75]. In this paper, the systematic development of the maintenance programme for the SLIMMER intervention was described, which might contribute to maintenance of the acquired healthy lifestyle.

## Conclusions

The IM protocol provided a useful framework to systematically develop a maintenance programme, incorporating insights from theory, literature, programme implementers, and participants. The study showed that planning a maintenance programme can build on existing implementation structures of the extensive programme. Future research should determine to what extent the maintenance programme contributes to sustained effects in participants of lifestyle interventions.

## Electronic supplementary material

Additional file 1: **Change objectives for the SLIMMER maintenance programme.** Table showing the behavioural outcomes, performance objectives, determinants and change objectives for participants of the SLIMMER maintenance programme. (PDF 296 KB)

Additional file 2: **Theoretical methods, definitions, parameters for use and practical applications for each behavioural determinant.** Table showing the theoretical methods, definition, parameters for use and practical application selected for the SLIMMER maintenance programme. (PDF 192 KB)

## Abbreviations

DPP: Diabetes prevention program
DPS: Diabetes prevention study
FGD: Focus group discussion
IM: Intervention mapping
RCT: Randomized controlled trial
SLIM: Study on lifestyle intervention and impaired glucose tolerance Maastricht
SLIMMER: SLIM iMplementation experience region Noord- en Oost-Gelderland
SDT: Self-determination theory
TPB: Theory of planned behaviour
TTM: Trans theoretical model
T2DM: Type 2 diabetes mellitus.
