# Supplementary material for: Using the intervention mapping protocol to develop a maintenance programme for the SLIMMER diabetes prevention intervention
Source: BMC Public Health. 2014 Oct 27;14:1108. doi: 10.1186/1471-2458-14-1108 (PMC4286928; doi:10.1186/1471-2458-14-1108)
Supplement: Supplementary file 1 — Additional file 1: Change objectives for the SLIMMER maintenance programme. Table showing the behavioural outcomes, performance objectives, determinants and change objectives for participants of the SLIMMER maintenance programme. (PDF 296 KB) [file 12889_2014_7366_MOESM1_ESM.pdf]

Additional file 1: Change objectives for the SLIMMER maintenance programme

**Behavioural outcome: SLIMMER participants maintain the acquired healthy diet independently**				
	Behavioural determinants			
**Performance objective:**	**Knowledge**	**Attitude**	**Subjective norm**	**Perceived behavioural control**
Comply with the Dutch dietary guidelines	Describe Dutch guidelines for healthy diet;<br>Explain why complying to Dutch guidelines healthy diet is important	Emphasize importance of a healthy diet	List other participants or persons from social environment who comply to guidelines healthy diet;<br>Mention the support they receive from their social environment when complying to guidelines healthy diet	Express confidence in handling negative social and environmental stimuli and obstructive thoughts which complicate compliance to guidelines healthy diet
Create social support to maintain healthy diet	Mention where they can find social support;<br>Explain why social support is important in maintaining a healthy diet	Convince others that social support is important	Mention the social support other participants or persons receive	Demonstrate that they can find social support when necessary
Identify situations that could be tempting to relapse	Describe situations in which they are tempted to relapse			Express confidence in resisting temptation to relapse
Compose action plans with realistic targets to maintain healthy diet	Explain importance of setting targets	Convince others that setting targets is important	List other participants or persons from social environment who have an action plan to maintain healthy diet;<br>Mention the support they receive from their social environment when composing an action plan	Demonstrate that they can set realistic targets and comply to these targets
Maintain monitoring of weight and diet	Explain why monitoring is important;<br>Describe how they can monitor	Emphasize importance of monitoring	List other participants or persons from social environment who monitor their	Express confidence in monitoring their weight and diet

	their weight and diet		weight and diet;<br>Mention the support they receive from their social environment when monitoring	
**Behavioural outcome: SLIMMER participants maintain the acquired healthy physical activity pattern independently**				
	Behavioural determinants			
**Performance objective:**	**Knowledge**	**Attitude**	**Subjective norm**	**Perceived behavioural control**
Comply with the Dutch norm for healthy physical activity	Describe Dutch norm for healthy physical activity;<br>Explain why complying to Dutch norm for healthy physical activity is important	Emphasize importance of physical activity	List other participants or persons from social environment who comply to physical activity norm;<br>Mention the support they receive from their social environment when complying to physical activity norm	Express confidence in handling negative social and environmental stimuli and obstructive thoughts which complicate compliance to physical activity norm
Create social support to maintain healthy physical activity pattern	Mention where they can find social support;<br>Explain why social support is important in maintaining physical activity	Convince others that social support is important	Mention the social support other participants or persons receive	Demonstrate that they can find social support when necessary
Identify situations that could be tempting to relapse	Describe situations in which they are tempted to relapse			Express confidence in resisting temptation to relapse
Compose action plan with realistic targets to maintain healthy physical activity pattern	Explain importance of setting targets	Convince others that setting targets is important	List other participants or persons from social environment who have an action plan to be physically active;<br>Mention the support they receive from their social environment when composing	Demonstrate that they can set realistic targets and comply to these targets

			an action plan	
Maintain monitoring of physical activity pattern	Explain why monitoring is important;<br>Describe how they can monitor their physical activity pattern	Emphasize importance of monitoring	List other participants or persons from social environment who monitor their physical activity pattern;<br>Mention the support they receive from their social environment when monitoring	Express confidence in monitoring their physical activity pattern
