# Supplementary material for: Using the intervention mapping protocol to develop a maintenance programme for the SLIMMER diabetes prevention intervention
Source: BMC Public Health. 2014 Oct 27;14:1108. doi: 10.1186/1471-2458-14-1108 (PMC4286928; doi:10.1186/1471-2458-14-1108)
Supplement: Supplementary file 2 — Additional file 2: Theoretical methods, definitions, parameters for use and practical applications for each behavioural determinant. Table showing the theoretical methods, definition, parameters for use and practical application selected for the SLIMMER maintenance programme. (PDF 192 KB) [file 12889_2014_7366_MOESM2_ESM.pdf]

Additional file 2: Theoretical methods, definitions, parameters for use and practical applications for each behavioural determinant

Behavioural Determinant	Theoretical method	Definition	Parameters for use	Practical application
General	Participation	Assuring high level engagement of the participants' group in problem solving, decision making, and change activities; with highest level being control by the participants' group	Requires willingness by health promoter to accept participants as having a high level of influence; requires participants' group to possess appropriate motivation and skills	Implementers and participants are included in the process of maintenance programme development
	Individualization	Providing opportunities for learners to have personal questions answered or instructions paced according to their individual progress	Personal communication that responds to a learner's needs	Participants can contact the project group through e-mail and phone with question when necessary
	Modeling	Providing an appropriate model being reinforced for desired action	Attention, remembrance, self-efficacy and skills, reinforcement of model, identification with model, coping model instead of mastery model	Trainers of sports clubs will present themselves as a role model who stimulates and motivates participants
	Feedback	Giving information to individuals and environmental agents regarding the extent to which they are accomplishing learning or performance, or the extent to which performance is having impact	Feedback needs to be individual, follow the behaviour in time and be specific	Physiotherapists and dieticians inform participants about their progression during return session
	Facilitation	Creating an environment that makes the action easier or reduces barriers to action	Requires real changes in the environment; identification of barriers and facilitators; power for making changes	By introducing participants to activities of local sports clubs, the barrier to join sports clubs is reduced
Knowledge	Advance organizers	Presenting an overview of material that enables the learner to activate relevant schemas so that new material	Schematic representations of content or guides to what is to be learned	Providing an online overview of activities of local facilitators of physical activity and healthy nutrition on

		can be associated		SLIMMER website
	Discussion	Encouraging consideration of a topic in an open informal debate	Listening to the learner to ensure that the correct schemas are activated	During return session participants discuss a relapse case and methods to prevent relapse
Habits	Implementation intentions	Prompting making if-then plans that link situational cues with responses that are effective in attaining goals or desired outcomes	Existing positive intention	Participants receive an action plan in which they formulate specific goals and ways to achieve them
	Planning coping responses	Getting the person to identify potential barriers and ways to overcome these	Identification of high-risk situations and practice of coping response	During concluding meeting participants identify situations in which they are tempted to relapse and think or ways to avoid relapse
Attitude	Self-reevaluation	Encouraging combing both cognitive and affective assessment of one’s self-image with and without an unhealthy behaviour	Stimulation of both cognitive and affective appraisal of self-image	During concluding meeting participant compare their current life and well-being to that before SLIMMER and realize they want to maintain their behaviour
	Direct experience	Encouraging a process whereby knowledge is created through the interpretation of experience	Rewarding outcomes from the individual’s experience with the behaviour or assurance that the individual can cope with and reframe negative outcomes	Participants try different sports during sports clinics which may change their attitudes about certain sports
	Elaboration	Stimulating the learner to add meaning to the information that is processed	Individuals with high motivation and cognitive ability	During return session, participants discuss how they feel about their behaviour change
Subjective norm	Resistance to social pressure	Stimulating building skills for resistance to social pressure	Commitment to earlier intention; relating intended behaviour to values; psychological inoculation against pressure	Participants learn how to deal with social pressure, for instance when they are at parties or dinners with friends

Perceived behavioural control	Self-monitoring of behaviour	Prompting the person to keep a record of specified behaviours	The monitoring must be of the specific behaviour; the data must be interpreted and used; the reward must be reinforcing to the individual	During concluding meeting, the importance of monitoring is explained and methods to monitor behaviour are provided. The importance of self-monitoring can be highlighted again during return session
	Goal setting	Prompting planning what the person will do, including a definition of goal-directed behaviours that result in target behaviour	Commitment to goal; goals that are challenging but achievable within the individual's skill level	During concluding meeting, participants set targets and make an action plan, which is added to the personal file of the participant
